# Where infectious diseases occur: restrictive measures and the concept of place

**DOI:** 10.1007/s40592-025-00237-2

**Published:** 2025-03-28

**Authors:** Diego S. Silva

**Affiliations:** Sydney Health Ethics, The University of Sydney, Sydney, Australia

**Keywords:** Coercion, Health security, Pandemics, Social justice, Equity, Public health ethics

## Abstract

Greater attention should be given to place when considering whether to, and how to, implement restrictive measures in response to infectious disease outbreaks and pandemics. Human beings cannot experience the world except in place, while place allows us to act ethically and in relation to other persons. Some have described place as a location with meaning to humans. Our individual and collective sense of meaning and identities are partly created in and by the places we live, while our sense of agency and security are shaped by them. Although taking the concept of place seriously is central to other disciplines and cultures, it is– with some notable exceptions– absent in the bioethics literature, including that of public health ethics. This paper attempts to outline how attending to the normative aspects of place can help explain some of our lingering COVID19-related trauma, as well as be used constructively in responding to future outbreaks when we cannot avoid the use of restrictive measures.

## Introduction

The use of restrictive measures in responding to infectious disease outbreaks, including pandemics, should take seriously the role of place in shaping who we are as persons. This is, undoubtedly, a modest claim that I will defend herein; still, place as a locum of moral inquiry is surprisingly absent in the public health ethics literature^1^. The moral valence of place– as something of intrinsic value - is often ignored within the analytic philosophy tradition, too, as Jeff Malpas notes, though it does have some history and traction in phenomenology and existentialism (2018). And yet the concept of place as worthy of investigation is inherent in other disciplines, including physics, geography, and architecture, while public health’s concern with One Health, ecology, and the built environment suggests improving the places where we live is critical for protecting and promoting health. The importance of place is also clear in many non-Anglo and non-European societies– for example, in Australia, the importance of Country, i.e., the nurturing relationship between the physical land and built environment with people, is imperative and prominent in the metaphysics and value systems of all the Aboriginal and Torres Strait Islander nations (Page and Memott 2021). So while my claim about the moral importance of place in responding to infectious disease outbreaks via restrictive measures is less common in the context of public health ethics, it’s likely to be obvious to many others outside our discipline.

To further sharpen my thesis, I want to propose that the trauma or lingering unease people are dealing with as the brunt of the COVID19 pandemic subsides for many of us^2^ can be partially explained by how our experiences of place were warped in light of the restrictive measures enacted during the pandemic. In turn, this suggests that we need to take seriously the effects of restrictive measures on our notion of place as we respond to future outbreaks. I begin by providing a rough description of place and its moral significance. By then discussing how place shapes and is shaped by human agency and our need for security, I provide some examples of how public health^3^ failed to account for the importance of place. I will end by providing an anecdote about what it means to take place seriously in responding to infectious diseases. I pre-emptively concede that many of the ideas described herein should be given much greater attention than I will provide. Given its brevity, the goal of this essay just is to sketch out the challenges and opportunities of attending to place in public health ethics, particularly as it relates to infectious diseases and restrictive measures, and to suggest there is much more work to be done in this domain.

Before doing that, I acknowledge that I’m assuming that public health’s use of restrictive measures– of which there are many different kinds– in responding to infectious disease outbreaks is coercive; but I’m also assuming these measures are, or could be, necessary in certain circumscribed situations. If public health law across jurisdictions permits the restriction of freedoms, primarily movement, in the context of emergencies, it’s the case that those preexisting freedoms are the *de facto* state-of-affairs. People placed under restrictive measures would likely experience them as coercive. Conceptually, restrictive measures are, indeed, coercive since not abiding by restrictions would lead to negative consequences that would otherwise not exist *but for* the state’s threats of what will happen if one doesn’t abide by public health orders. Again, none of that suggests that restrictive measures are morally wrong; in this essay, I’ll remain agnostic as to their rightness or wrongness.

## Place: what is it?

One helpful and encapsulating way of understanding the notion of place is as, to borrow from Yi-Fu Tuan’s description, a location with meaning to humans (2021). It is where external physical structures shape our behaviour, and it’s where we act and exercise our agency. Place is the locus of experience, and in the process, we create our sense of identity, our self-narratives, and therefore, our meaning. As Malpas notes, the ability to experience ourselves and others is constitutive of– among other factors– place; *we cannot experience except in place*, and hence, to live in places and move through space just is part of what it means to be human (2018). Malpas goes on to note that places are bounded and contained but this doesn’t mean movement is simply constrained, but rather, that it facilitates movement by allowing us to know when we move into places with different meanings, meanings that are imbued partly or largely by humans (ibid.). For example, from inside one’s apartment, where we’re mostly free to act as we’d like, we move into a common area like a hallway with certain rules and etiquette, and then onto the world outside our building with yet a different set of rules of behaviour.

In addition to place’s role in human metaphysics and epistemology, place helps shape and ground our understanding of ethics. Eckenwiler notes that “…we should resist a reductive, decontextualized view of individuals as the units of (moral and political) concern, for we cannot properly be conceived apart from our embeddedness in social relations and geographically, atmospherically identifiable locations in which we become and endure” (2013). Bridging the feminist and ecological traditions, Eckenwiler notes that place helps makes sense not only of who we are, i.e., relational beings relative to each other and to place simultaneously, but that it’s where we do ethics and where we enact our norms and values. In what she calls ethical placemaking, Eckenwiler claims that “…societies’ [have] obligations to provide the conditions in which people can care and be cared for” (2016). For those of us working in public health ethics, this may seem trivially or obviously true; after all, there’s a long history of taking seriously the social, economic, and political determinants of health, and thus, surely built and natural environments are inherently part of such determinants. Yet place’s taken-for-grantedness– its inherent implicitness– means that it’s often missing in plain view when we respond to infectious diseases. At best and most generous, place remains morally undertheorized and primarily the domain of those working on issues related to non-communicable diseases, but even here it is rarely the case that place is treated as a concept worth taking seriously in-and-of-itself.

## The place of agency and security

How we understand the relationship between place and ethics would require, among other things, some analysis of the ways place shapes and is shaped by other morally significance concepts, including principles and values. Agency and security are two such concepts, which I raise here to then demonstrate how place matters in the context of outbreaks and pandemics.

By agency I mean something like the ability to act and to be, and be held, responsible for those actions. Agency presupposes some kinds and amounts of freedoms, including some freedom to move within and between places. To be sure, our physical environments promote certain movements, deters others, and prohibits certain actions outright. Here’s a trivial and classic example: the cement paths in a park directs us to move along those paths, while park benches with arm rests discourage rough sleeping of homeless persons, while trees mean we can’t go through them but only around them. But we’ve all likely seen parks where the shortest path from point A to B is across the grass and people will walk that path to the extent of killing the grass and creating a dirt rut. In other words, we aren’t merely passive subjects of our external environments but shape them, too. As such, we move, act, and are agents in place, and it’s through those actions in relation to physical space and with others that we give our lives meaning and help build places for ourselves and others, i.e., locations that take on meaning and become places through our– collective– actions.

The relationship between place and security is also of critical importance. Generally speaking, to secure *x* means ensuring that *x* will exist for one’s or someone’s use sometime in the future. For example, if you’ve made a reservation at your favourite restaurant, you’re ensuring that you’ll have a table available at some specified future moment in time. Securing a table means other future patrons are prohibited from using it during the time I’m meant to be there. There are conditions, of course– perhaps you need to show up no later than 15 min after the reserved time otherwise the table will go to someone else. While some things are worth incurring high costs to secure– e.g., life– most others will only be secured contingently. And borrowing from Jonathan Herington’s work, therein lies one of the reasons security is morally interesting and vexing, since securing something reliably means precluding others’ actions, and securing something practically absolutely– or to a high degree– means precluding others from interfering practically absolutely (2012). The moral cost of securing is, therefore, high. And as such, states only secure– or should only secure– life’s most important things. In practice, sometimes what we deem *prima facie* worth securing are physical places given their significance or necessity for life, or their particularly important shared symbolic meaning. For example, meaningful access to housing, which we then transform into our homes, would seem to be something worth securing for people, as would the right to enter and exit one’s country (Silva 2022).

## Pandemics and place

Our security and agency were dramatically altered during the COVID19 pandemic, in part, because of the lack of attention to the moral importance of place when using restrictive measures to curb the spread of the virus. This claim seems not only intuitively plausible, but there is evidence to suggest it’s true, too: the use of lockdowns and the policing of borders are two such examples.

First, during lockdowns many people were confined to their homes or abided by other severe restrictions on their movements– including how far they could travel and the reasons for travel– when they ventured outside their homes, assuming people were not suffering from homelessness. The experience of lockdowns varied by, among other factors, ethnicity and socioeconomic status (SES) and, by extension, where one lived (Bann et al. 2021). For example, in Sydney, Australia, western suburbs– i.e., neighbourhoods– were policed more stringently than the rest of the city, which included the use of the Australian Defence Forces patrolling alongside police in areas of the city where most residents were non-white and of lower SES (Xiao 2021). In 2021, when the virus was purportedly spreading in Western Sydney at higher rates than the rest of the city, the Minister of Health in the state of New South Wales claimed that “…people from other backgrounds… don’t really give great consideration to what they do in terms of its impact on the rest of the community” (McMahon and Robertson 2021). The Minister’s imputing a sense of disregard among a people of a particular geographical region– ‘geographically’ different here both in terms of where they lived in the city and the fact that many were born outside of Australia– disregarded how many in those neighbourhoods lived in multigenerational homes where the primary breadwinners had to care for both elderly and young family members, and where employment was tied to services that couldn’t be performed in-home yet were essential for others to remain safely in lockdowns, e.g., supermarket workers or food delivery drivers. Eckenwiler’s notion of ethical placemaking is important here in diagnosing our moral rebuke with this story by calling attention to the way people relate not in abstraction, but in concrete and material ways in particular places. The racism, which at best one could charitably describe as inadvertent, in the enforcement of lockdown orders in Sydney isn’t just about place, but rather place is a key part of explaining how racism can hide behind public health goals and procedures. Geographic location was used to justify the separation of ‘us’ from ‘them’; to borrow from Nurcan Akbulut and Oliver Razum’s work (2022), physical place was weaponized as a means of ‘othering’ a particular disenfranchised group of people.

The second category of example is the ways borders were controlled throughout the height of the COVID19 pandemic. Australian citizens, for example, described feeling abandoned given how difficult the federal and state governments made it to return home (Capper 2021; Mao 2021); here, by home, I mean not only the physical country of Australia but also coming back to their physical domiciles. In their qualitative studies, Bridget Haire and Jane Williams found that participants who underwent hotel quarantine when returning from abroad felt that they were owed something by the Australian state and public for being in quarantine for two weeks, e.g., food, internet, and access to healthcare services, which is in keeping with the principle or value of reciprocity (Williams et al. 2022; Pahlman et al. 2023). Yet a few months after the start of the pandemic, persons returning home had to pay $3000 out-of-pocket to stay in mandatory hotel quarantine. The states refused to fully fund the system claiming, in short, that Australians who hadn’t left before or during the pandemic shouldn’t foot the bill for other people’s– other Australians’ - decisions to travel and then return home. Australians who returned felt deeply aggrieved for several reasons: many hotels were not fit for purpose, e.g., windows didn’t open, rooms were too small for families returning with kids, rooms lacked proper sunlight, etc. Moreover, their right of return as citizens were being restricted beyond what could reasonably be deemed necessary for public health reasons. The most notable case was when Australians who were in India during the initial phases of the Delta outbreak were prohibited to return to Australia for a period of two weeks on threat of imprisonment (Silva 2022). The harm done unto these persons goes beyond, I would claim, the immediate challenges associated with restricted movement and travel, toward something much more fundamental– it impeded and challenged their identities as Australians, developing a sense of being second-class citizens, by altering the places that provide meaning and security, while often literally *placing* them in locations devoid of meaning and the security to which they were normally accustomed and morally entailed.

What these two examples suggest is that our narratives of ourselves and our identities are challenged abruptly and externally through restrictive measures that alter our experiences of, and relations to, place. This is part of why restrictive measures are so difficult to accept for so many people: they disrupt our notion of security and agency and hence, our meanings and identities. To reiterate what I wrote at the outset, this isn’t to say that restrictive measures are morally impermissible during outbreaks, but rather that the harm associated with them are not limited to the duration of time our free movement is impeded– it lingers. There’s something deeper at play, and it includes altering how we view ourselves in those places that give us meaning. Moreover, this altered sense of place, and subsequently agency and security, isn’t limited to merely our physical homes; it includes public places, since neighbourhoods also provide an element of meaning– just think of some of your favourite cafes, pubs, theatres, etc. and our warm memories of these places when we’re not in them. Therefore, lockdowns, as one kind of restrictive measure, don’t just mean we’re spending most of our time in our homes in ways that are artificial and beyond what they can reasonably provide for us, but that being deprived of enjoying public places– intrinsically, but also given how our relationship with others are created and function outside our homes, i.e., as part of ethical placemaking– is damaging, too.

## How being attentive to place helps

I want to conclude by suggesting that taking seriously place can have a positive effect when restrictive measures are instituted to try to contain infectious diseases. In previous research, when I was analysing how the harm principle was applied in the contexts of persons with severe mental illnesses and tuberculosis, I interviewed public health workers as well as staff from two psychiatric hospitals in Toronto, Canada (Silva 2019). A nurse from one of the psychiatric hospitals told the following story about when an inpatient was diagnosed with a respiratory infectious disease:

…we had to shut down a part of the unit so there were no admissions there…there was an area of the unit that could be shut off so other people wouldn’t be exposed to the infection but it ended up being a compromise in that a couple of other beds were closed down [and] as a result of that [we gave] her the space. So there’s an example where in order to meet her needs and to protect the rest of the people on the unit, she was given more space to wander but on the whole it means fewer people can be admitted there in general…. I mean the only other option would have been to keep her in one little single room, which would have been really cruel I think for someone like her. I don’t know that she could have understood that (Silva 2013).

As the nurse notes, the situation was somewhat serendipitous, but the staff gave attention and care to the safety and wellbeing not only of others on the ward, but the person suffering from the infection and psychiatric condition, too, by being attentive to place and its role in mitigating harm. It was also a resource intensive response; scaling-up a similar response would require a large financial commitment on the part of the state or hospital. Regardless, it’s an instructive anecdote whereby place was used to *lessen* the effects of a restrictive measure. It suggests that thinking seriously about the places where public health restrictions occur, and brainstorming creatively about solutions that explicitly take them into account, can have potentially powerful remedial consequences on people’s lives. 

As researchers in public health ethics, the various ways place can be conceptualised and its role in how we think about public health challenges and policy should not be restricted merely to questions about coercion, restrictive measures, and infectious diseases. The scope of future research is wide and includes questions around the application of place to specific challenges - e.g., disability, the climate emergency, the destruction of civilian infrastructure and homes as intentional acts of war, etc. - as well as the analysis of place vis-a-vis the preexisting applied ethics architecture, i.e., place as it relates to previously and well-articulated values and mid-level principles in the literature. Building off the work of Lisa Eckenwiler and colleagues, as well as making concrete efforts to engage with existing literature about place from other disciplines, augurs well for important and impactful future public health ethics research.^4^

## Data Availability

No datasets were generated or analysed during the current study.

## Footnotes

1 Albeit with the prominent exception being the work of Lisa Eckenwiler and a small handful of her colleagues– see Eckenwiler 2013; 2016; 2020; Haverkamp and Eckenwiler 2022; Eckenwiler and Wild 2022.

2 The spread of SARS-CoV2, and the associated morbidity and mortality, have– at best– subsided but have not gone away. Persons with various disabilities, for example, are still experiencing grave effects and their plight shouldn’t be minimized, e.g, long-COVID. This is doubly true given the tendency to ignore or look past persons with disabilities who are often treated as less valuable in our efforts to move past the pandemic (Scully 2023).

3 Who or what constitutes ‘public health’ is itself a vague but important question. For the purposes of this essay, ‘public health’ denotes those decision-makers who have authority to implement restrictive measures at various levels of government, while acknowledging that their use of restrictive measures could be helped or hindered by broader political or other contextual features.

4 Thank you to the reviewers for nudging me to briefly consider the broader scope and future research implications of place in public health ethics. 
